# Comparison of Outcomes of Haploidentical Peripheral Blood Stem Cell Transplantation Supported by Third-Party Cord Blood Versus Human Leukocyte Antigen-Matched Sibling Peripheral Blood Stem Cell Transplantation in Hematologic Malignancy Patients

**DOI:** 10.3389/fonc.2022.922120

**Published:** 2022-07-14

**Authors:** Tingting Cheng, Yan Chen, Yi Liu, Xia Ma, Cong Zeng, Xu Chen, Shiyu Wang, Yajing Xu

**Affiliations:** ^1^ Department of Hematology, Xiangya Hospital, Central South University, Changsha, China; ^2^ National Clinical Research Center for Geriatric Diseases, (Xiangya Hospital), Changsha, China; ^3^ Hunan Hematologic Neoplasms Clinical Medical Research Center, Xiangya Hospital, Central South University, Changsha, China; ^4^ National Clinical Research Center for Hematologic Diseases The First Affiliated Hospital of Soochow University, Suzhou, China

**Keywords:** haploidentical donor, peripheral blood, cord blood, stem cell transplantation, HLA-matched sibling donor, hematologic malignancy

## Abstract

Recent studies have shown that haploidentical hematopoietic stem cell transplantation supported by third-party cord blood (haplo-cord-HSCT) results in rapid hematopoietic recovery, low incidences of graft-versus-host disease (GVHD), and relapse of hematologic malignancies. However, few reports on haploidentical peripheral blood stem cell transplantation supported by third-party cord blood (haplo-cord-PBSCT) have been published. To evaluate the outcomes of patients who underwent haplo-cord-PBSCT or human leukocyte antigen (HLA)-matched sibling donor peripheral blood stem cell transplantation (MSD-PBSCT), we retrospectively reviewed the clinical data of patients with hematologic malignancies who underwent haplo-cord-PBSCT (n = 93) or MSD-PBSCT (n = 72) in our hospital from March 2017 to December 2020. In the haplo-cord-PBSCT and MSD-PBSCT groups, the median time for neutrophil and platelet engraftment was 13 vs. 12 days (*p* = 0.07) and 16 vs. 13 days (*p* = 0.06), respectively. The 30-day cumulative incidences of neutrophil engraftment were 100.0% and 98.6% (*p* = 0.12). The 100-day cumulative incidences of platelet engraftment were 96.8% and 98.6% (*p* = 0.01). The 100-day cumulative incidences of grade II–IV and grade III–IV acute GVHD were 29.1% vs. 23.6% (*p* = 0.42) and 9.7% vs. 4.2% (*p* = 0.18). The cumulative incidences of total and moderate/severe chronic GVHD at 1 year were 26.5% vs. 17.4% and 8.1% vs. 4.5%, respectively, and at 3 years were 34.7% vs. 34.3% (*p* = 0.60) and 13.6% vs. 10.6% (*p* = 0.49), respectively. The cumulative incidences of relapse at 1 year were 9.3% and 7.2% and at 3 years were 17.0% and 17.0% (*p* = 0.98). Non-relapse mortality (NRM) at 1 year was 14.6% and 8.6% and at 3 years was 17.4% and 8.6% (*p* = 0.13) in two groups. The probabilities of overall survival (OS), disease-free survival (DFS), and GVHD-free/relapse-free survival (GRFS) at 1 year were 81.7% vs. 88.6%, 76.1% vs. 84.2%, and 71.7% vs. 79.7%, respectively, and at 3 years were 78.7% vs. 79.0%, 65.6% vs. 74.4%, and 55.5% vs. 63.6%, respectively, in the corresponding group, *p* > 0.05. In conclusion, for patients with acute myeloid leukemia/myelodysplastic syndrome (AML/MDS) and acute lymphoid leukemia (ALL), haplo-cord-PBSCT results in similar outcomes compared with MSD-PBSCT, and it may be a valid alternative transplantation method.

## Introduction

Allogeneic hematopoietic stem cell transplantation (allo-HSCT) is an important choice for treating hematologic malignancies. The development of haploidentical hematopoietic stem cell transplantation (haplo-HSCT) has not only contributed to solving the issues with the shortage of donors but also brought hope for patients urgently needing hematopoietic stem cell transplantation but lacking human leukocyte antigen (HLA)-matched donors. The data from the worldwide Network for Blood and Marrow Transplantation Group show that haplo-HSCT procedures have increased rapidly in recent years (1, 2). According to the data of Chinese Bone Marrow Transplantation Registration (CBMTR) for 2019, haploidentical transplantation accounted for 60% of all allo-HSCT, showing an increasing yearly trend (3). In China, haploidentical donors have become the main allo-HSCT donor sources. Three major haplo-HSCT modalities have been defined: T-cell depletion (TCD) transplantation with posttransplant high-dose cyclophosphamide (PTCY), granulocyte colony-stimulating factor (G-CSF)-primed bone marrow (BM) plus peripheral blood (PB) graft, and anti-thymocyte globulin (ATG)-based regimens, called “Beijing Protocol,” and *in vitro* TCD protocol (3). The application of the aforementioned schemes has reduced the incidence of graft-versus-host disease (GVHD) (4, 5). In China, the “Beijing Protocol” was implemented in approximately 94% of the haplo-HSCT, based on previous data (3). Earlier studies confirmed that haplo-HSCT using the “Beijing Protocol” had similar overall survival (OS) and disease-free survival (DFS) to those of HSCT from HLA-matched sibling donors (MSDs) or HLA-matched unrelated donors (MUDs) (6, 7). Furthermore, haplo-HSCT showed a stronger graft-versus-leukemia (GVL) effect than MSD-HSCT, especially in high-risk, refractory, and pretransplantation measurable residual-positive acute leukemia (AL) (6–9). The stem cell sources of haplo-HSCT are various, including BM, PB, or a combination of them (BM + PB). PB is the main stem cell source in both the United States and Europe (1). However, in China, BM + PB constitutes 59% of the haplo-HSCT cases, while simple PB or BM transplantation accounts for less than 30% (3). Little data exist about PB stem cell transplantation (PBSCT).

Haplo-HSCT has achieved promising results in the treatment of hematologic malignancies. However, the delayed and unstable immune reconstitution and the incidence of GVHD increase the rate of non-relapse mortality (NRM) in haplo-HSCT (10, 11). Umbilical cord blood (UCB) transplantation (UCBT) is another option for HSCT due to its rapid graft acquisition and low incidence of GVHD and relapse (12, 13), while UCB stem cells are less prone to delay and failure of engraftment (14, 15). Recent studies reported that haplo-HSCT combined with third-party cord blood accelerated engraftment reduced the incidence of GVHD and the recurrence of hematologic malignancies, which was superior to either haploidentical or UCB transplantation alone (16–18). To investigate the effects of haplo-cord-HSCT, some studies have compared this transplantation scheme with HLA-matched transplantation and obtained similar OS, DFS, NRM, and GVHD incidence rates (19–21). However, the stem cell sources examined in previous studies have predominantly been BM + PB, whereas very few studies have investigated haploidentical PBSCT supported by third-party cord blood (haplo-cord-PBSCT). To further explore the effect of haplo-cord-PBSCT, we retrospectively compared this transplantation scheme with MSD-PBSCT.

## Materials and Methods

### Patients

From March 2017 to December 2020, patients who received allo-HSCT at Xiangya Hospital, Central South University (Changsha, Hunan, China), were included in this study if they met the following criteria: 1) they were older than 14 years and were diagnosed with AL or myelodysplastic syndrome (MDS), 2) received haplo-cord-PBSCT or MSD-PBSCT 3) received modified Bu/Cy myeloablative preparative regimen. This research was approved by the Ethics Committee of Xiangya Hospital. Written informed consent was obtained from all patients or their legal guardians.

### Preparative Regimen

Modified Bu/Cy regimen was used in all patients: cytarabine (Ara-C) (haplo-cord-PBSCT group, 4 g/m^2^/day, from days −8 to −7; MSD-PBSCT group, 2 g/m^2^/day, from days −8 to −7), busulfan (Bu) (3.2 mg/kg/day, from days −6 to −4), cyclophosphamide (CTX) (1.8 g/m^2^/day, from days −3 to −2), and semustine (250 mg/m^2^, day −2). r-ATG (rabbit anti-human thymocyte immunoglobulin) was administered at 2.5 mg/kg/day (days −3 to −1) in the haplo-cord-PBSCT group.

### Human Leukocyte Antigen Typing and Donor Selection

High-resolution techniques were used for HLA-A, HLA-B, HLA-C, HLA-DRB1, and HLA-DQB1 typing to select haploidentical donors. All patients receiving haplo-cord-PBSCT were tested for the presence of donor-specific anti-HLA antibodies (DSAs), including class I and class II HLA antibodies. The median fluorescence intensity (MFI) of DSA in all patients was lower than 4,000, and they did not receive treatment before transplantation. Haploidentical donors were selected on the basis of HLA typing, DSA testing, age, gender, health status, and willingness to donate. The UCB unit was selected based on the high resolution of HLA-A, HLA-B, HLA-DRB1, and HLA-DQB1. The following criteria for cord blood unit selection were applied: 1) at least 5/10 matched-HLA loci and 2) total nucleated cells not less than 1 × 10^7^/kg of the recipient’s body weight after thawing. Blood type-matched cord blood was preferred at an equal level of HLA-type matching.

### Graft Collection and Infusion

G-CSF (7.5–10 μg/kg/day) was used to mobilize PB stem cells 4 days before HSCT. In the haplo-cord-PBSCT group, the haploidentical cells were infused on the first day, followed by cord blood mononuclear cell (MNC) infusion (1 × 10^7^/kg) on the second day. The interval between the cord blood transfusion and the end of PBSC transfusion was at least 12 h.

### Graft-Versus-Host Disease Prophylaxis and Treatment

All transplantation recipients received cyclosporine A (CsA), mycophenolate mofetil (MMF), and short-term methotrexate (MTX) as GVHD prophylaxis.

CsA was given to the haplo-cord-PBSCT recipients by continuous infusion at 2.5 mg/kg/day from day −9 until the patients could switch to oral intake after the recovery of their gastrointestinal function, with a target blood concentration ranging from 200 to 250 μg/L. MTX at 15 mg/m^2^ was administered on day +1, and MTX at 10 mg/m^2^ was given on days +3, +6, and +11. MMF was given at 0.5 g PO twice per day from day −9; the dose was halved from day +30 until day +45 to day +60 if no GVHD occurred.

CsA was given to MSD-PBSCT recipients by continuous infusion at 2.5 mg/kg/day from day −1 until patients could switch to oral intake, with a target blood concentration ranging from 200 to 250 μg/L. MTX at 15 mg/m^2^ was administered on day +1, and MTX at 10 mg/m^2^ was given on days +3 and +6. MMF was given at 0.5 g PO twice per day from day −1 until engraftment.

The first-line treatment for grade II–IV acute GVHD (aGVHD) was methylprednisolone infusion at 2 mg/kg/day. Second-line therapy such as basiliximab was used in patients with methylprednisolone intolerance or poor response.

### Supportive Care and Posttransplantation Evaluation

Patients started gut decontamination with gentamicin and nystatin prior to conditioning 3–5 days before HSCT. Prophylactic antibiotics and antifungal and antiviral drugs were used during the preparative regimen and immunosuppression period; sulfamethoxazole was administrated to prevent *Pneumocystis carinii* infection. Bone marrow aspirations were performed at least once a month in the first 6 months after transplantation and then every 1–1.5 months in the second 6 months, every 1.5–2 months in the second year, and every 2–3 months thereafter to evaluate the remission status and chimerism.

### Endpoints and Definitions

The diagnosis of acute lymphoid leukemia (ALL), acute myeloid leukemia (AML), and MDS was based on the WHO criteria (22). Risk stratifications were performed with reference to the National Comprehensive Cancer Network (NCCN) guidelines (23, 24) and the International Prognostic Scoring System (IPSS) (25). Patients with refractory/relapsed disease were included in the high-risk group. Granulocyte recovery was defined as the first day of three consecutive days when absolute neutrophil count (ANC) >0.5 × 10^9^/L. Platelet recovery was defined as the first day of platelet counts >20 × 10^9^/L without transfusion support for seven consecutive days. The diagnosis was made, and GVHD grading criteria were implemented with reference to earlier publications (26–28). Relapse was defined on the basis of BM histology analysis with more than 5% blasts or extramedullary relapse. NRM was defined as death due to any cause without previous disease progression or relapse. OS was calculated from the date of transplantation to the date of death due to any cause, and surviving patients were censored at the last follow-up examination. DFS was defined as survival in continuous complete remission without relapse. GVHD-free/relapse-free survival (GRFS) was calculated from the date of HSCT to the date of events that included grade III–IV aGVHD, chronic GVHD (cGVHD) requiring systemic therapy, relapse, or death.

### Statistical Analysis

The χ^2^ or Fisher’s exact test was used to compare categorical variables. A non-parametric test (Mann–Whitney U‐test) was employed to compare continuous variables. One- and three-year OS or DFS was calculated using the Kaplan–Meier outcome curve and compared by log-rank test. Considering the competing risks, the cumulative incidence rate (CIR) of engraftment, aGVHD, cGVHD, NRM, and relapse were calculated by the Gray test. Competing events were defined as follows: death due to any cause without engraftment as the competing event for engraftment; relapse, engraftment failure, or death as the competing event for GVHD; relapse as the competing event for NRM; and death as the competing event for relapse. Multivariate analyses of transplantation-related covariates affecting survival were determined using the Cox proportional hazard model. All variables presented in **Table 1** were included in a univariate analysis (**Supplementary Table**). The forced factor (haplo-cord-PBSCT *vs.* MSD-PBSCT) and variables with *p* < 0.10 were included in further multivariate analysis. The SPSS26.0 software package (SPSS, Chicago, IL, USA) was used for data analysis. R software (version 4.1.1; https://www.r-project.org/) was utilized for competing risk analysis. All tests were two-sided, and *p* < 0.05 was considered to indicate statistically significant differences.

**Table 1:** Patient and graft characteristics.

Characteristics	Haplo-cord PBSCT	MSD-PBSCT	*p*-Value
Total patients	93	72	
Age, years, median (range)	32 (14–56)	36 (14–53)	0.20
Gender, n (%)			0.49
Male	49 (52.7)	34 (47.2)	
Female	44 (47.3)	38 (52.8)	
Diagnosis, n (%)			0.21
AML/MDS	53 (57.0)	48 (66.7)	
ALL	40 (43.0)	24 (33.3)	
Risk stratification, n (%)			
AML/MDS			0.06
Intermediate	19 (35.8)	26 (54.2)	
High	34 (64.2)	22 (45.8)	
ALL			0.34
Standard	9 (22.5)	8 (33.3)	
High	31 (77.5)	16 (66.7)	
All patients, n (%)			0.02*
Standard/intermediate	28 (30.1)	34 (47.2)	
High	65 (69.9)	38 (52.8)	
Disease status before HSCT, n (%)			0.06
CR1	65 (69.9)	46 (63.9)	
≧CR2	17 (18.3)	8 (12.5)	
Non-CR	11 (11.8)	18 (23.6)	
Donor–patient sex matched, n (%)			0.72
Matched	40 (43.0)	29 (40.3)	
Mismatched	53 (57.0)	43 (59.7)	
Donor–patient blood type matched, n (%)			0.23
Matched	59 (63.4)	52 (72.2)	
Mismatched	34 (36.6)	20 (27.8)	
HLA compatibility, n (%)			
5/10	53 (57.0)	–	
6/10	23 (24.7)	–	
7/10	9 (9.7)	–	
8/10	6 (6.5)	–	
9/10	2 (2.1)	–	
10/10	–	72 (100.0)	
Infused MNCs, ×10^8^/kg (range)	10.30 (4.63~19.30)	8.20 (3.78~18.49)	0.000*
Infused CD34^+^ cells, ×10^6^/kg (range)	5.74 (1.61~14.12)	4.57 (2.01~13.10)	0.005*

haplo-cord-PBSCT, haploidentical donor peripheral blood stem cell transplantation supported by third-party cord blood; MSD-PBSCT, HLA-matched sibling donor peripheral blood stem cell transplantation; AML, acute myeloid leukemia; ALL, acute lymphoid leukemia; MDS, myelodysplastic syndrome; CR1, first complete remission; CR2, second complete remission; HLA, human leukocyte antigen; MNC, mononuclear cells.

*p < 0.05.

## Results

### Patients’ Characteristics

A number of 165 patients who underwent haplo-cord-PBSCT (n = 93, 56.4%) or MSD-PBSCT (n = 72, 43.6%) from March 2017 to December 2020 were enrolled in this study. The characteristics of the patients are displayed in **Table 1**. There were 53 (57.0%) patients with AML/MDS and 40 (43.0%) patients with ALL in the haplo-cord-PBSCT group, and 48 (66.7%) patients with AML/MDS and 24 (33.3%) patients with ALL in the MSD-PBSCT group. The patients with high-risk features in the haplo-cord-PBSCT group were higher in number than those in the MSD-PBSCT group (69.9% *vs.* 52.8%, *p* = 0.02).

The last follow-up date was January 31, 2022. The median follow-up time in the haplo-cord- and MSD-PBSCT groups was 26 (3–59) and 28 (2–60) months, respectively.

### Donor and Graft Characteristics

**Table 1** shows the characteristics of donor and graft. In the haplo-cord-PBSCT group, 31 (33.3%) haploidentical related donors were patients’ parents, 24 (25.8%) were patients’ children, and 38 (40.9%) were patients’ siblings. The median doses of infused MNCs and CD34^+^ cells in the haplo-cord-PBSCT group were 10.30 × 10^8^/kg (range, 4.63–19.30) and 5.74 × 10^6^/kg (range, 1.61–14.12), respectively. The number of UCB MNCs infused on the second day was 1 × 10^7^/kg.

For the MSD-PBSCT group, the median MNC content was 8.20 × 10^8^/kg (range, 3.78–18.49), and the median CD34^+^ cell content was 4.57 × 10^6^/kg (range, 2.01–13.10), which were lower than those in the haplo-cord-HSCT group (MNC, *p* = 0.000; CD34^+^ cell, *p* = 0.005).

### Hematopoietic Recovery and Engraftment

Full donor chimerism was achieved in all patients by day 30 posttransplantation, except for two patients in the haplo-cord-PBSCT group who died of infection before platelet engraftment on day +76 and day +81, as well as with the exception of one primary graft failure (PGF) patient who died of sepsis on day +63 after MSD-PBSCT. No cord blood chimerism or mixed chimerism was established in the haplo-cord-PBST group.

**Figure 1:**
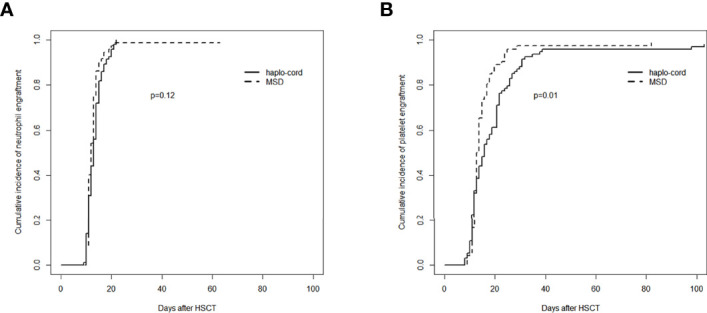
Assessment of hematopoietic recovery after transplantation in haplo-cord group and MSD group: **(A)** cumulative incidence of neutrophil engraftment and **(B)** cumulative incidence of platelet engraftment. MSD, matched sibling donor.

In the haplo-cord and MSD-PBSCT groups, the median time to neutrophil engraftment was 13 days (range, 9–22) and 12 days (range, 10–21), respectively (*p* = 0.07). The cumulative incidence of neutrophil engraftment at day 30 was 100.0% and 98.6% (95% CI: 84.1–99.9) in the two groups (*p* = 0.12, **Figure 1A**). Multivariate analysis revealed no significant difference in the neutrophil engraftment between the two groups (hazard ratio (HR) = 0.797, 95% CI: 0.607–1.047, *p* = 0.100), but recipient age >40 years was confirmed as an independent risk factor for neutrophil engraftment (HR = 0.737, 95% CI: 0.556–0.979, *p* = 0.035, **Table 2**).

**Table 2:** Multivariate analyses for transplant outcomes.

Outcomes	Multivariate analysis		
	HR	95% CI	*p*
**Neutrophil engraftment**			
Group (haplo-cord vs. MSD)	0.797	0.607~1.047	0.100
Age (>40 vs. ≤40)	0.737	0.556~0.979	0.035*
**Platelet engraftment**			
Group (haplo-cord vs. MSD)	0.665	0.481~0.921	0.014*
Age (>40 vs. ≤40)	0.767	0.564~1.043	0.091
**aGVHD (II~IV grade)**			
Group (haplo-cord vs. MSD)	1.280	0.701~2.330	0.420
**aGVHD (III~IV grade)**			
Group (haplo-cord vs. MSD)	2.089	0.535~8.150	0.290
Diagnose (AML/MDS vs. ALL)	0.327	0.094~1.140	0.079
**cGVHD**			
Group (haplo-cord vs. MSD)	1.170	0.687~2.000	0.560
Donor-patient gender (mismatch vs. matched)	1.730	0.969~3.100	0.064
**cGVHD (moderate/severe)**			
Group (haplo-cord vs. MSD)	1.546	0.611~3.910	0.360
Donor-patient blood type (mismatch vs. matched)	0.336	0.097~0.160	0.085
**NRM**			
Group (haplo-cord vs. MSD)	1.970	0.761~5.090	0.160
Infused MNC cells (≧median vs. <median)	2.540	0.989~6.520	0.053
**Relapse**			
Group (haplo-cord vs. MSD)	0.947	0.386~2.320	0.910
Risk classification (high vs. others)	2.058	0.694~6.100	0.190
Disease status before HSCT (CR1 vs. others)	0.612	0.251~1.490	0.280
Infused CD34^+^ cells (≧median vs. <median)	0.459	0.189~1.120	0.087
**OS**			
Group (haplo-cord vs. MSD)	1.153	0.555~2.394	0.703
Risk classification (high vs. others)	2.287	0.945~5.531	0.067
Disease status before HSCT (CR1 vs. others)	0.689	0.316~1.501	0.348
Infused MNC cells (≧median vs. <median)	1.687	0.782~3.640	0.182
Infused CD34^+^ cells (≧median vs. <median)	0.387	0.177~0.848	0.018*
**DFS**			
Group (haplo-cord vs. MSD)	1.454	0.766~2.761	0.253
Diagnose (AML/MDS vs. ALL)	0.812	0.434~1.517	0.514
Risk classification (high vs. others)	1.857	0.881~3.917	0.104
Disease status before HSCT (CR1 vs. others)	0.559	0.299~1.048	0.070
Infused CD34^+^ cells (≧median vs. <median)	0.451	0.235~0.867	0.017*
**GRFS**			
Group (haplo-cord vs. MSD)	1.316	0.774~2.238	0.311
Diagnose (AML/MDS vs. ALL)	0.694	0.410~1.175	0.174
Infused CD34^+^ cells (≧median vs. <median)	0.509	0.295~0.878	0.015*

aGVHD, acute graft versus host disease; cGVHD, chronic graft versus host disease; NRM, non-relapse mortality; OS, overall survival; DFS, disease-free survival; GRFS, GVHD-free/relapse-free survival.

*p < 0.05.

The median time to platelet engraftment was 16 days (range, 8–103) and 13 days (range, 9–82) in the haplo-cord and MSD-PBSCT groups, respectively (*p* = 0.06). The cumulative incidence of platelet engraftment at day 100 in the haplo-cord-PBSCT group (96.8% [95% CI: 88.7%–99.1%]) was significantly lower than that in the MSD-PBSCT group (98.6% [95% CI: 71.0%–99.9%]) (*p* = 0.01, **Figure 1B**). On multivariate analysis, inferior platelet engraftment was associated with haplo-cord-PBSCT (HR = 0.665, 95% CI: 0.481–0.921, *p* = 0.014, **Table 2**).

### Graft-Versus-Host Disease

**Figure 2:**
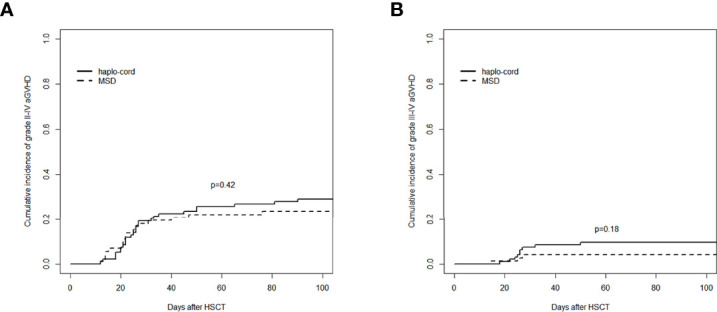
Assessment of cumulative incidence of aGVHD in haplo-cord group and MSD group: **(A)** grade II–IV aGVHD and **(B)** grade III–IV aGVHD. aGVHD, acute graft-versus-host disease; MSD, matched sibling donor.

**Figure 3:**
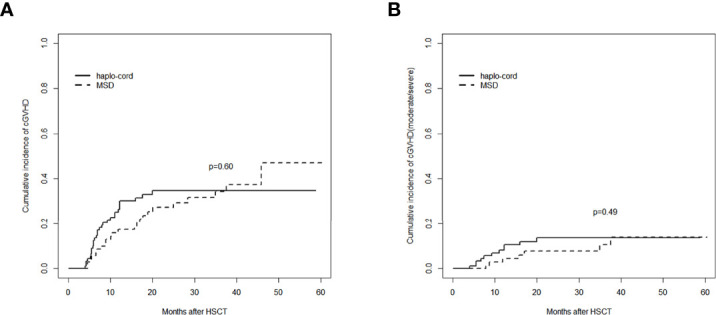
Assessment of cumulative incidence of cGVHD in haplo-cord group and MSD group: **(A)** all grades of cGVHD and **(B)** cGVHD (moderate/severe). cGVHD, chronic graft-versus-host disease; MSD, matched sibling donor.

The cumulative incidence of aGVHD (grade II–IV) at day 100 after haplo-cord-PBSCT was 29.1% (95% CI: 20.2%–38.5%), comparable to that of the MSD-PBSCT group (23.6% [95% CI: 14.5%–34.0%], *p* = 0.42, **Figure 2A**). The cumulative incidence of aGVHD (grade III–IV) at day 100 was 9.7% (95% CI: 4.7%–16.7%) in the haplo-cord-PBSCT group versus 4.2% (95% CI: 1.1%–10.7%) in the MSD-HSCT group (*p* = 0.18, **Figure 2B**). The cumulative incidences of cGVHD at 1 year were 26.5% (95% CI: 17.7–36.1%) and 17.4% (95% CI: 9.5%–27.2%) (*p* = 0.60) and at 3 years were 34.7% (95% CI: 24.5%–45.1%) and 34.3% (95% CI: 22.2%–46.8%) (*p* = 0.60, **Figure 3A**). The cumulative incidences of moderate/severe cGVHD at 1 year were 8.1% (95% CI: 3.5%–15.0%) and 4.5% (95% CI: 1.2%–11.4%) (*p* = 0.49) and at 3 years were 13.6% (95% CI: 7.1%–22.3%) and 10.6% (95% CI: 4.0%–20.8%) (*p* = 0.49, **Figure 3B**) for the haplo-cord and MSD-PBSCT groups, respectively. Our multivariate analysis (**Table 2**) showed also no significant differences in the risk of grade II–IV aGVHD (HR = 1.280, 95% CI: 0.701–2.330, *p* = 0.420), grade III–IV aGVHD (HR = 2.089, 95% CI: 0.535–8.150, *p* = 0.290), cGVHD (HR = 1.170, 95% CI: 0.687–2.000, *p* = 0.560), and moderate/severe cGVHD (HR = 1.546, 95% CI: 0.611–3.910, *p* = 0.360) in the haplo-cord-PBSCT group versus the MSD-PBSCT group.

### Non-Relapse Mortality

**Figure 4:**
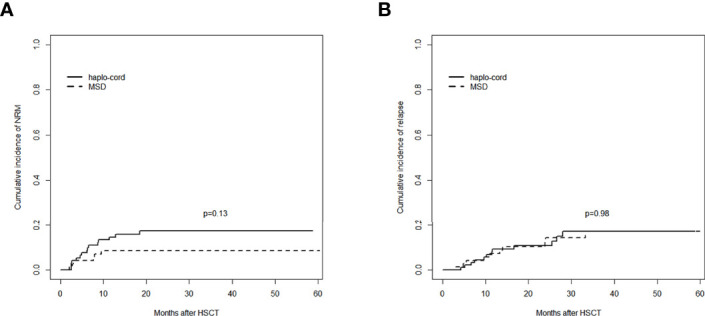
Comparisons of outcomes between the haplo-cord group and the MSD group: **(A)** cumulative incidence of NRM and **(B)** cumulative incidence of relapse. MSD, matched sibling donor; NRM, non-relapse mortality.

The 1-year cumulative incidence of NRM in the haplo-cord-PBSCT group was 14.6% (95% CI: 8.2%–22.7%), whereas it was 8.6% (95% CI: 3.5%–16.6%) in the MSD-PBSCT group (*p* = 0.13). The 3-year cumulative incidences of NRM in these two groups were 17.4% (95% CI: 10.2%–26.2%) and 8.6% (95% CI: 3.5%–16.6%) (*p* = 0.13, **Figure 4A**).

The causes of NRM included severe GVHD and severe infections such as pneumonia or sepsis. Fifteen NRM cases occurred in the haplo-cord-PBSCT group, whereas six cases occurred in the MSD-PBSCT group (*p* = 0.14). The multivariate analysis results revealed (**Table 2**) no difference in the risk of NRM (HR = 1.970, 95% CI: 0.761–5.090, *p* = 0.160) between the haplo-cord-PBSCT and the MSD-PBSCT groups.

### Relapse

The 1- and 3-year cumulative incidences of relapse in the haplo-cord-PBSCT group were 9.3% (95% CI: 4.3%–16.6%) and 17.0% (95% CI: 9.0%–27.3%), respectively, whereas they were 7.2% (95% CI: 2.6%–14.9%) and 17.0% (95% CI: 8.4%–28.2%), respectively, in the MSD-PBSCT group *(p* = 0.98, **Figure 4B**). The multivariate analysis (**Table 2**) showed no significant difference in the risk of relapse (HR = 0.947, 95% CI: 0.386–2.320, *p* = 0.910) between the haplo-cord and MSD-PBSCT groups.

### Survival

**Figure 5:**
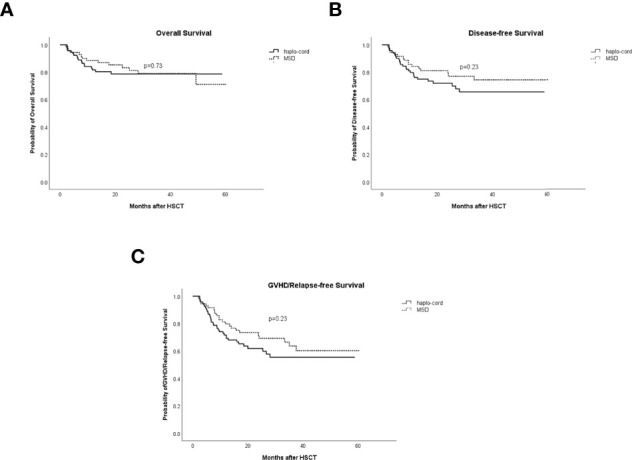
Comparisons of survival rates in haplo-cord group and MSD group. **(A)** Overall survival (OS). **(B)** Disease-free survival (DFS). **(C)** GVHD/relapse-free survival (GRFS). MSD, matched sibling donor; GVHD, graft-versus-host disease.

The 1- and 3-year OS in the haplo-cord-PBSCT group was 81.7% (95% CI: 73.9%–90.2%) and 78.7% (95% CI: 70.3%–88.1%), respectively, compared with 88.6% (95% CI: 81.4–96.4%) and 79.0% (95% CI: 69.3%–90.2%), in the MSD-PBSCT group (*p* = 0.73, **Figure 5A**). The multivariate analysis results showed no significant difference in OS (HR = 1.153, 95% CI: 0.555–2.394, *p* = 0.703) between the two groups. Inferior OS was associated with a low dose of CD34^+^ cells (HR = 2.584, 95% CI: 1.179–5.650, *p* = 0.018).

The 1- and 3-year DFS in the haplo-cord- and MSD-PBSCT was similar at 76.1% (95% CI: 67.7%–85.6%) versus 84.2% (95% CI: 76.0%–93.2%) (*p* = 0.23) and 65.6% (95% CI: 55.4%–77.8%) versus 74.4% (95% CI: 64.0%–86.5%) (*p* = 0.23, **Figure 5B**), respectively. Our multivariate analysis data revealed no significant difference in DFS (HR = 1.454, 95% CI: 0.766–2.761, *p* = 0.253) between the two groups. A lower dose of infused CD34^+^ cells was the only risk factor for DFS identified in our multivariate analysis (HR = 2.217, 95% CI: 1.195–4.255, *p* = 0.017, **Table 2**).

The 1- and 3-year GRFS was 71.7% (95% CI: 62.8%–81.8%) versus 79.7% (95% CI: 70.8%–89.8%) (*p* = 0.23) and 55.5% (95% CI: 44.9%–68.5%) versus 63.6% (95% CI: 51.9%–77.9%), respectively, in the haplo-cord-PBSCT and MSD-PBSCT groups (*p* = 0.23, **Figure 5C**). Multivariate analysis showed no significant difference in GRFS (HR = 1.316, 95% CI: 0.774–2.238, *p* = 0.311) between the haplo-cord and MSD-PBSCT groups; the infusion with a low amount of CD34^+^ cells was the risk factor affecting GRFS (HR = 1.965, 95% CI: 1.139–3.390, *p* = 0.015, **Table 2**).

## Discussion

The application of the “Beijing Protocol” and PTCY has contributed to an improvement in the effect of haploidentical transplantation applied in the treatment of patients with hematologic malignancies. However, high NRM incidence still occurs in haplo-HSCT due to delayed, unstable immune reconstitution and high GVHD incidence (10, 11). PBSCT has the advantages of rapid engraftment, low incidence of relapse, and greater convenience and ease of collection as compared with BM; however, it is often accompanied by a high GVHD incidence (29, 30). UCBT has a low GVHD incidence but a high incidence of delayed engraftment, and its failure is caused by the limited stem cell number of UCB, which increases the risk of early death (14, 15). Here, we designed haploidentical PBSCT supported by third-party cord blood by combining the characteristics of these two types of grafts. We found that this method could achieve outcomes similar to those of HLA-MSD PBSCT.

In the present analysis, we observed similar median time and cumulative incidence of neutrophil engraftment at day 30 in the haplo-cord and MSD-PBSCT groups. The median time of platelet engraftment was 16 days in the haplo-cord-PBSCT group, which was similar to that in the MSD-PBSCT group. The cumulative incidence of platelet engraftment at 100 days after haplo-cord-PBSCT was significantly lower than that after MSD-PBSCT but similar to that achieved in previous studies of haplo-cord-HSCT (21, 31). Bashey et al. (32) established that the median time of platelet engraftment was 31 days, and the cumulative engraftment rate at day 100 was 84.0% after haploidentical PBSCT alone with PTCY. In another investigation, Zhao et al. (33) reported that the median time of platelet engraftment in haplo-PBSCT with the use of ATG/G-CSF was 16 days, and the cumulative engraftment rate at day 100 was 90.5%. We observed higher cumulative incidence of platelet engraftment in the haplo-cord-PBSCT group than the ones obtained in the aforementioned previous research work, indicating that haploidentical transplantation supported by cord blood may promote hematopoietic recovery. In our study, full haploidentical chimerism without evidence of UCB or mixed chimerism was achieved in all survival patients who underwent haplo-cord-PBSCT, which was different from the findings of other studies (31, 34). This outcome may be attributed to the low number of UCB MNCs (1 × 10^7^/kg) that were infused at least 12 h after the end of the haploid graft infusion.

Our data showed similar cumulative incidences of grade II–IV aGVHD, grade III–IV aGVHD, cGVHD, and moderate/severe cGVHD between the haplo-cord and MSD-PBSCT groups. These outcomes may be due to the addition of ATG and UCB in the haplo-cord-PBSCT group, which prevented GVHD. Previous studies indicated that UCB-Treg exhibits predominantly naïve (CD45RAhi) and almost no activated or memory subsets, which allowed UCB-Treg to demonstrate a superior proliferative capability to PB-Treg (35). The adoptive transfer of UCB-derived Tregs reduced the risk of acute GVHD (36). In concordance, we did not observe a higher incidence rate of GVHD in the haplo-cord-PBSCT group compared with the MSD-PBSCT group. Ma et al. (30) analyzed the outcomes of PBSCT alone with ATG/G-CSF and found that the cumulative incidences of grade II–IV aGVHD at day 100, grade III–IV aGVHD at day 100, cGVHD at one year, and moderate/severe cGVHD at 1 year were 29.9%, 7.5%, 54.9%, and 17.4%, respectively, which were higher than those in our haplo-cord-PBSCT group, especially in cGVHD incidence. A possible explanation of these results could be that UCB may regulate the hematopoietic microenvironment and reduce immune rejection (37). However, compared with haplo-cord-PBSCT in our study, other studies showed lower cumulative incidences of 100-day aGVHD (20.0%–27.0%) and 1-year cGVHD (20.0%–27.0%) in haplo-cord-HSCT (17, 19, 20). One reason may be that our ATG dosage (7.5 mg/kg) was lower than that of other studies (10 mg/kg) (17, 20). From another perspective, we only used PB as haploid graft, whereas other researchers utilized mainly PB + BM. Notably, G-CSF administration significantly decreased the expression of adhesion molecules involved in GVHD on naïve CD4^+^ and CD8^+^ T cells in BM grafts and led to polarization of BM naïve CD4^+^ and CD8^+^ T cells from Th1 to Th2 phenotype, which may lead to a lower incidence of GVHD (38, 39). However, the incidence rates of grade III–IV aGVHD and moderate/severe cGVHD in our haplo-cord-PBSCT group were lower than those of other studies of haplo-cord-HSCT (20).

Here, we found no significant difference in the cumulative incidence of NRM between the haplo-cord-PBSCT and MSD-PBSCT groups. Salvatore et al. (10) retrospectively analyzed data for the period 2011–2015 obtained from the European Society of Blood and Marrow Transplantation. These scientists established that the NRM in haploidentical transplantation was higher than that of HLA-matched sibling transplantation. Haplo-HSCT supported by UCB reduced the incidence of GVHD and promoted hematopoietic recovery; it lowered the risk of infection associated with early neutropenia and the bleeding associated with platelet deficiency. Moreover, we presumed that the low ATG dosage in our investigation also decreased the risk of cytomegalovirus/Epstein–Barr virus (CMV/EBV) infection (40). Therefore, no high NRM incidence was observed in the haplo-cord-PBSCT group. However, the NRM of haplo-cord-PBSCT in the present study was similar to that established in another study on haplo-PBSCT alone (41), which may have been due to the high proportion of patients with high-risk features in our study.

We observed a similar 3-year cumulative incidence of relapse between the haplo-cord and MSD-PBSCT groups (17.0% vs. 17.0%). In MDS patients, Ke et al. (20) reported a similar 2-year cumulative incidence of relapse between haplo-cord-HSCT and MSD-HSCT (12.0% vs. 14.0%). Furthermore, Lu et al. (31) also found no significant difference in the 2-year cumulative relapse incidence between haplo-cord-HSCT and MSD-HSCT (14.2% vs. 12.0%), which was consistent with our conclusion. However, the results of another study suggested that haplo-cord-HSCT had a stronger GVL effect and lower 2-year cumulative incidence of relapse than MUD-HSCT (7.5% vs. 21.9%) (21). Wang et al. (42) also revealed that the additional infusion of UCB with a haplo graft reduced the relapse rate of refractory AL due to the graft-versus-graft (GVG) effect, similar to that of double UCB. However, a similar effect was not observed in our study, which may be related to the fact that more high-risk patients were included in the haplo-cord-PBSCT group than in the MSD-PBSCT group (*p* = 0.02).

Similar OS, DFS, and GRFS in the haplo-cord and MSD-PBSCT groups were obtained in the present study, which is consistent with the results of previous studies (19, 20, 31). In addition, we confirmed that OS, DFS, and GRFS were related to the dosage of CD34^+^ cells. Törlén et al. also found that the CD34^+^ dose was associated with survival after allogeneic transplantation for AML/MDS (43).

Our study has some limitations. First, it was a retrospective cohort study, and some imbalances might have existed, although multivariate analysis has adjusted the imbalanced factors. Second, because of the small sample size, we did not compare patients with different diseases and different risk stratification categories. Thus, the sample size needs to be increased to analyze subgroups and design a prospective randomized study for further research. Furthermore, the mechanism of action of UCB in haploidentical transplantation needs to be additionally explored.

In summary, the findings of this study indicate that haploidentical PBSCT supported by third-party cord blood results in OS and cumulative incidences of GVHD, NRM, and relapse, similar to those of MSD PBSCT, although the patients with high-risk features in the haplo-cord-PBSCT group were more than those in the MSD-PBSCT group. Our study also confirmed the safety and efficacy of haplo-cord-PBSCT. In terms of stem cell collection, we were able to avoid the risks of anesthesia, cross-infection, and local hemorrhage during BM harvest. Furthermore, the rapid acquisition of UCB and the simplicity and convenience of PBSC collection were more easily accepted by donors. Therefore, in patients with hematologic malignancies, especially those with high-risk features, haplo-cord-PBSCT provides a viable and promising therapeutic option.

## Data Availability Statement

The raw data supporting the conclusions of this article will be made available by the authors, without undue reservation.

## Ethics Statement

Written informed consent was obtained from the individual(s), and minor(s)’ legal guardian/next of kin, for the publication of any potentially identifiable images or data included in this article.

## Author Contributions

YX and TC conceived and designed the study and helped to draft the manuscript. YC, YL, XM, and CZ performed the data collection. TC, SW, and XC performed the statistical analysis. All authors read and critically revised the manuscript for intellectual content and approved the final manuscript.

## Funding

This work was supported by the National Natural Science Foundation of China (No. 81974002) and Translational Research Grant of NCRCH (No. 2021WWC02).

## Conflict of Interest

The authors declare that the research was conducted in the absence of any commercial or financial relationships that could be construed as a potential conflict of interest.

## Publisher’s Note

All claims expressed in this article are solely those of the authors and do not necessarily represent those of their affiliated organizations, or those of the publisher, the editors and the reviewers. Any product that may be evaluated in this article, or claim that may be made by its manufacturer, is not guaranteed or endorsed by the publisher.
