# Supplementary material for: Comparison of Outcomes of Haploidentical Peripheral Blood Stem Cell Transplantation Supported by Third-Party Cord Blood Versus Human Leukocyte Antigen-Matched Sibling Peripheral Blood Stem Cell Transplantation in Hematologic Malignancy Patients
Source: Front Oncol. 2022 Jul 14;12:922120. doi: 10.3389/fonc.2022.922120 (PMC9331161; doi:10.3389/fonc.2022.922120)
Supplement: Supplementary file 1 [file Table_1.docx]

**TableS1.** Cumulative incidence of Neutrophil and Platelet recovery in Univariate Analysis

| **Variable** | **Platelet engraftment at d100** | ***P* value** | **Neutrophil engraftment at d30** | ***P* value** |
| --- | --- | --- | --- | --- |
| **Group** |  | 0.013* |  | 0.120 |
| MSD | 98.6% |  | 98.6% |  |
| Haplo-cord | 96.8% |  | 100.0% |  |
| **Gender** |  | 0.146 |  | 0.331 |
| Female | 98.8% |  | 100.0% |  |
| Male | 96.4% |  | 98.8% |  |
| **Age** |  | 0.089* |  | 0.083* |
| ≦40 years | 99.0% |  | 100.0% |  |
| >40 years | 95.0% |  | 98.3% |  |
| **Diagnose** |  | 0.836 |  | 0.349 |
| AML/MDS | 97.0% |  | 99.0% |  |
| ALL | 98.4% |  | 100.0% |  |
| **Risk classification** |  | 0.193 |  | 0.826 |
| High | 98.1% |  | 100.0% |  |
| others | 96.8% |  | 98.4% |  |
| **Disease Status pre- HSCT** |  | 0.213 |  | 0.518 |
| CR1 | 97.3% |  | 99.1% |  |
| Others | 98.1% |  | 100.0% |  |
| **Donor-patient gender match** |  | 0.130 |  | 0.865 |
| mismatched | 99.0% |  | 99.0% |  |
| matched | 95.6% |  | 100.0% |  |
| **Donor-patient blood**  **type match** |  | 0.991 |  | 0.384 |
| mismatched | 98.1% |  | 100.0% |  |
| matched | 97.3% |  | 99.1% |  |
| **Infused MNC cells** |  | 0.130 |  | 0.629 |
| ≧median | 95.2% |  | 98.8% |  |
| <median | 100.0% |  | 100.0% |  |
| **Infused CD34^+^ cells** |  | 0.497 |  | 0.457 |
| ≧median | 97.3% |  | 100.0% |  |
| <median | 98.1% |  | 98.8% |  |

*indicate *P* < 0.10

Abbreviations: MSD: HLA-matched sibling donor; AML, acute myeloid leukemia; ALL, acute lymphoid leukemia; MDS, myelodysplastic syndrome; HSCT, hematopoietic stem cell transplantation; CR1, first complete remission; MNC, mononuclear cells.

**TableS2.** Cumulative incidence of aGVHD and cGVHD in Univariate Analysis

| **Variable** | **aGVHD(grade II-IV) at d100** | ***P* value** | **aGVHD (grade III-IV) at d100** | ***P***  **value** | **cGVHD at 3 years** | ***P***  **value** | **cGVHD (moderate/severe) at 3 years** | ***P***  **value** |
| --- | --- | --- | --- | --- | --- | --- | --- | --- |
| **Group** |  | 0.420 |  | 0.182 |  | 0.602 |  | 0.489 |
| MSD | 23.6% |  | 4.2% |  | 34.3% |  | 10.6% |  |
| Haplo-cord | 29.1% |  | 9.7% |  | 34.7% |  | 13.6% |  |
| **Gender** |  | 0.932 |  | 0.970 |  | 0.132 |  | 0.514 |
| Female | 25.6% |  | 7.3% |  | 41.7% |  | 15.4% |  |
| Male | 27.7% |  | 7.2% |  | 28.1% |  | 9.3% |  |
| **Age** |  | 0.829 |  | 0.406 |  | 0.843 |  | 0.208 |
| ≦40 years | 26.7% |  | 8.6% |  | 36.3% |  | 15.4% |  |
| >40 years | 26.7% |  | 5.0% |  | 33.5% |  | 7.1% |  |
| **Diagnose** |  | 0.104 |  | 0.040* |  | 0.128 |  | 0.354 |
| AML/MDS | 22.8% |  | 12.5% |  | 40.8% |  | 10.5% |  |
| ALL | 32.8% |  | 4.0% |  | 26.9% |  | 15.3% |  |
| **Risk classification** |  | 0.174 |  | 0.117 |  | 0.486 |  | 0.338 |
| High | 30.1% |  | 9.7% |  | 31.5% |  | 10.1% |  |
| others | 21.0% |  | 3.2% |  | 39.4% |  | 15.7% |  |
| **Disease Status pre- HSCT** |  | 0.846 |  | 0.500 |  | 0.284 |  | 0.560 |
| CR1 | 27.0% |  | 6.3% |  | 37.2% |  | 12.2% |  |
| Others | 25.9% |  | 9.3% |  | 30.4% |  | 12.3% |  |
| **Donor-patient gender match** |  | 0.452 |  | 0.516 |  | 0.057* |  | 0.147 |
| mismatched | 28.1% |  | 8.3% |  | 39.7% |  | 14.3% |  |
| matched | 24.6% |  | 5.8% |  | 28.2% |  | 9.4% |  |
| **Donor-patient blood**  **type match** |  | 0.528 |  | 0.229 |  | 0.855 |  | 0.079* |
| mismatched | 24.1% |  | 3.7% |  | 34.1% |  | 6.7% |  |
| matched | 27.9% |  | 9.0% |  | 35.8% |  | 15.7% |  |
| **Infused MNC cells** |  | 0.887 |  | 0.235 |  | 0.743 |  | 0.509 |
| ≧median | 26.5% |  | 9.6% |  | 35.0% |  | 12.0% |  |
| <median | 26.8% |  | 4.9% |  | 34.9% |  | 12.6% |  |
| **Infused CD34^+^ cells** |  | 0.847 |  | 0.564 |  | 0.183 |  | 0.615 |
| ≧median | 26.5% |  | 8.4% |  | 40.3% |  | 9.6% |  |
| <median | 26.8% |  | 6.1% |  | 29.7% |  | 15.0% |  |

*indicate *P* < 0.10

Abbreviations: aGVHD, acute graft-versus-host disease; cGVHD, chronic graft-versus-host disease; MSD: HLA-matched sibling donor; AML, acute myeloid leukemia; ALL, acute lymphoid leukemia; MDS, myelodysplastic syndrome; HSCT, hematopoietic stem cell transplantation; CR1, first complete remission; MNC, mononuclear cells.

**TableS3.** Cumulative incidence of NRM and relapse in Univariate Analysis

| **Variable** | **Relapse at 3years** | ***P* value** | **NRM at 3 years** | ***P* value** |
| --- | --- | --- | --- | --- |
| **Group** |  | 0.977 |  | 0.129 |
| MSD | 17.0% |  | 8.6% |  |
| Haplo-cord | 17.0% |  | 17.4% |  |
| **Gender** |  | 0.122 |  | 0.554 |
| Female | 21.6% |  | 11.6% |  |
| Male | 12.6% |  | 15.2% |  |
| **Age** |  | 0.990 |  | 0.225 |
| ≦40 years | 16.8% |  | 11.3% |  |
| >40 years | 17.5% |  | 17.2% |  |
| **Diagnose** |  | 0.146 |  | 0.431 |
| AML/MDS | 12.7% |  | 11.7% |  |
| ALL | 23.0% |  | 16.2% |  |
| **Risk classification** |  | 0.080* |  | 0.156 |
| High | 22.0% |  | 16.2% |  |
| others | 9.8% |  | 8.7% |  |
| **Disease Status pre- HSCT** |  | 0.082* |  | 0.141 |
| CR1 | 13.3% |  | 10.3% |  |
| Others | 24.3% |  | 19.9% |  |
| **Donor-patient gender match** |  | 0.112 |  | 0.958 |
| mismatched | 11.0% |  | 13.6% |  |
| matched | 23.9% |  | 13.4% |  |
| **Donor-patient blood**  **type match** |  | 0.762 |  | 0.121 |
| mismatched | 14.5% |  | 19.6% |  |
| matched | 19.1% |  | 10.3% |  |
| **Infused MNC cells** |  | 0.758 |  | 0.039* |
| ≧median | 16.0% |  | 18.9% |  |
| <median | 18.2% |  | 7.8% |  |
| **Infused CD34^+^ cells** |  | 0.060* |  | 0.103 |
| ≧median | 12.4% |  | 18.0% |  |
| <median | 21.3% |  | 8.9% |  |

*indicate *P* < 0.10

Abbreviations: NRM: nonrelapse mortality; MSD: HLA-matched sibling donor; AML, acute myeloid leukemia; ALL, acute lymphoid leukemia; MDS, myelodysplastic syndrome; HSCT, hematopoietic stem cell transplantation; CR1, first complete remission; MNC, mononuclear cells.

**TableS4.** OS.DFS and GRFS in Univariate Analysis

| **Variable** | **OS at 3 years** | ***P* value** | **DFS at 3 years** | ***P* value** | **GRFS at 3years** | ***P* value** |
| --- | --- | --- | --- | --- | --- | --- |
| **Group** |  | 0.725 |  | 0.229 |  | 0.228 |
| MSD | 79.0% |  | 74.4% |  | 63.6% |  |
| Haplo-cord | 78.7% |  | 65.6% |  | 55.5% |  |
| **Gender** |  | 0.585 |  | 0.470 |  | 0.344 |
| Female | 75.6% |  | 66.8% |  | 54.4% |  |
| Male | 80.9% |  | 72.2% |  | 63.7% |  |
| **Age** |  | 0.601 |  | 0.965 |  | 0.541 |
| ≦40 years | 78.8% |  | 68.9% |  | 55.9% |  |
| >40 years | 77.7% |  | 74.9% |  | 69.2% |  |
| **Diagnose** |  | 0.528 |  | 0.087* |  | 0.045* |
| AML/MDS | 80.2% |  | 75.7% |  | 48.7% |  |
| ALL | 75.2% |  | 68.3% |  | 62.9% |  |
| **Risk classification** |  | 0.028* |  | 0.016* |  | 0.177 |
| High | 71.7% |  | 73.4% |  | 61.5% |  |
| others | 87.9% |  | 81.5% |  | 54.9% |  |
| **Disease Status pre- HSCT** |  | 0.024* |  | 0.012* |  | 0.114 |
| CR1 | 83.4% |  | 76.5% |  | 65.1% |  |
| Others | 67.9% |  | 55.8% |  | 47.2% |  |
| **Donor-patient gender match** |  | 0.604 |  | 0.212 |  | 0.778 |
| mismatched | 78.8% |  | 75.4% |  | 63.4% |  |
| matched | 77.8% |  | 62.7% |  | 54.4% |  |
| **Donor-patient blood type match** |  | 0.130 |  | 0.299 |  | 0.858 |
| mismatched | 71.5% |  | 65.9% |  | 61.2% |  |
| matched | 81.3% |  | 70.6% |  | 56.2% |  |
| **Infused MNC cells** |  | 0.058* |  | 0.151 |  | 0.279 |
| ≧median | 73.5% |  | 65.1% |  | 54.2% |  |
| <median | 83.2% |  | 74.0% |  | 63.8% |  |
| **Infused CD34^+^ cells** |  | 0.005* |  | 0.006* |  | 0.006* |
| ≧median | 86.4% |  | 78.6% |  | 70.6% |  |
| <median | 70.0% |  | 60.6% |  | 47.9% |  |

*indicate *P* < 0.10

Abbreviations: OS: overall survival; DFS: disease-free survival; GRFS: GVHD-free/relapse-free survival; MSD: HLA-matched sibling donor; AML, acute myeloid leukemia; ALL, acute lymphoid leukemia; MDS, myelodysplastic syndrome; HSCT, hematopoietic stem cell transplantation; CR1, first complete remission; MNC, mononuclear cells.
